# Incidence and Risk Factors of Thromboembolism with Multiple Myeloma in the Presence of Death as a Competing Risk: An Empirical Comparison of Statistical Methodologies

**DOI:** 10.3390/healthcare4010016

**Published:** 2016-02-26

**Authors:** Joshua D. Brown, Val R. Adams

**Affiliations:** Institute for Pharmaceutical Outcomes and Policy, Department of Pharmacy Practice and Science, College of Pharmacy, University of Kentucky, Lexington, KY 40536, USA; val.adams@uky.edu

**Keywords:** venous thromboembolism, multiple myeloma, competing risks

## Abstract

Multiple myeloma (MM) has an inherent high risk of thromboembolic events associated with patient as well as disease- and treatment-related factors. Previous studies have assessed the association of MM-related thromboembolism using “traditional” Kaplan–Meier (KM) and/or Cox proportional hazard (PH) regression. In the presence of high incidence of death, as would be the case in cancer patients with advanced age, these statistical models will produce bias estimates. Instead, a competing risk framework should be used. This study assessed the baseline patient demographic and clinical characteristics associated with MM-related thromboembolism and compared the cumulative incidence and the measures of association obtained using each statistical approach. The cumulative incidence of thromboembolism was 9.2% using the competing risk framework and nearly 12% using the KM approach. Bias in the measures of covariate risk associations was highest for factors related to risk of death such as increased age (75% bias) and severe liver disease (50%) for the Cox PH model compared to the competing risk model. These results show that correct specification of statistical techniques can have a large impact on the results obtained.

## 1. Introduction

Compared to the general population, individuals with cancer are at 4 to 7 times higher risk of developing a venous thromboembolism (VTE) [1,2,3,4]. Malignancy induces a prothrombotic state which includes activation of the coagulation cascade, increase in pro-inflammatory cytokines, as well as inhibition of natural anticoagulants and is further exacerbated by cancer treatment and surgery [5,6,7]. Other risk factors for VTE in cancer include the site and stage of the tumor, older age, prior history of clots, and comorbidities [6,8,9,10]. Although at an already increased risk of death from cancer, VTE carries a substantial risk of mortality with clotting events accounting for up to 10% of all deaths in patients with cancer [11,12,13].

Multiple myeloma (MM) has one of the highest risks of thrombosis among all cancers due to disease-related pathological changes and treatment [7,14]. Thalidomide and lenalidomide (IMIDs) are well known to be associated with increased risk of thrombosis [15], especially when combined with high-dose steroids and other chemotherapy, with incidence approaching 25% in some studies [16,17,18,19]. Other common MM treatments include proteasome inhibitors (PIs; bortezomib, carfilzomib) and cytotoxic therapies (cyclophosphamide, melphalan, others), which have been shown to have a lower, though still increased, risk of VTE compared to IMIDs [20]. Other disease-related factors with potential to increase thrombotic risk include use of central venous catheters (CVC), erythropoietin agents, hospitalization, and infection [6,10,21,22,23,24,25,26]. Due to this inherent increased risk of thrombosis with MM, guidelines U.S. guidelines recommend routine thromboprophylaxis with low-molecular weight heparins (LMWH) or aspirin [27,28].

These previous studies have assessed VTE risk in MM during randomized-controlled trials (RCTs) comparing alternative treatment strategies or in small observational utilizing “traditional” Kaplan–Meier and/or Cox proportional hazard models. In these statistical frameworks, death is considered a censoring event. This violates a basic assumption that censoring events and the outcome of interest must be independent of each other, meaning that censoring is uninformative. However, given that the occurrence of death prevents the outcome from ever occurring, death *competes* with the outcome and should not be considered a censoring event [29]. Instead, a competing risks framework should be used which treats death as a separate outcome. A study by Ay *et al.* [29] compared traditional methods to competing risk methods and found significant bias when ignoring death as a competing risk with VTE incidence during cancer. They observed that the bias in the cumulative incidence of thromboembolism is a function of the incidence of the competing risk. This study did not look at individual factors that may be associated with thromboembolism and how estimates of relative association may be impacted by these methods.

The objective of this study was to determine the one-year incidence of thrombotic events in MM and to assess the association of baseline characteristics and thrombosis in these patients while comparing traditional time-to-event methods with competing risk methods. This study compared risk associations between the two statistical frameworks as applied to this specific cancer type and calculated the bias that would be observed with “traditional” methods.

## 2. Materials and Methods

### 2.1. Data Source

This was a retrospective cohort study utilizing Truven Health Analytics MarketScan Commercial Claims and Encounters and Medicare Supplemental databases during the years 2008–2013. The MarketScan data are administrative claims data including medical diagnostic and procedural billing information and pharmacy fill records for those with commercial insurance linked to demographic and insurance enrollment information for each individual. The data include information regarding medical encounters for nearly 40 million persons each year and are representative of the commercially insured population in the U.S.

### 2.2. Patient Inclusion

We included patients with at least two separate claims with a diagnosis of MM (ICD-9: 203.0x) at least 14 days apart. For further inclusion, subjects were required to have a minimum of 6 months of continuous medical and pharmacy insurance coverage prior to the first MM diagnosis and be at least 18 years or older at diagnosis. Subjects also could not have a previous diagnosis of another cancer or a thrombotic outcome event during the 6-month, pre-index period.

### 2.3. Subject Characteristics

Age was assessed on the MM index date and gender was linked from the enrollment file. The Charlson Comorbidity Index was used to assess comorbidity burden based on the ICD-9-CM coding algorithm [30] and the total score was further categorized by 0, 1–2, 3–4, and 5+ groups with individual comorbidities also reported. Additional comorbidities of interest were also assessed during the 6 month pre-index period, including thrombocytosis (ICD-9: 238.71, 289.9), leukocytosis (ICD-9: 288.5), anemia (ICD-9: 280.x–285.x), obesity (ICD-9: 278.01–278.03, V85.3, V85.4), hypocoagulopathies (ICD-9: 286.x) hypercoagulopathies (ICD-9: 289.81–289.82), thrombocytopenia (ICD-9: 238.71, 289.9), and leukopenia (ICD-9: 288.5x).

### 2.4. Outcome Events

DVT and PE events were assessed based on previously published ICD-9-CM algorithms [31,32,33]. PVT was identified by ICD-9-CM code 452.x and AT identified by ICD-9-CM code 444.x. Date of death was based on discharge status codes on hospital or hospice records and loss to follow-up occurred when continuous insurance coverage ended during the follow-up period or follow-up terminated at the end of the data (December 2013). All other individuals were censored after one year of follow-up. If a thrombosis occurred on the same day as death, the event was recorded as the thrombosis as it was the main outcome of interest in this study. Subjects were followed until one of the following occurred: (1) a thrombosis event; (2) death; (3) loss to follow-up; or (4) end of the 1 year study period.

### 2.5. Survival Analysis

Person time was calculated correcting for the differential follow-up of each subject. The incidence rate of thrombosis was reported as the rate per 1000 person-years. The association of thrombosis with baseline demographic and clinical characteristics at diagnosis was assessed using a competing risks regression model. In this model the dependent outcome has three levels, 0 = censored, 1 = thrombosis, and 2 = death. Subdistribution hazard ratios (HR) and their 95% confidence intervals (CI) for the association between each baseline covariate and thrombotic events were estimated for each baseline covariate included in the model. For demonstration of the selection of statistical methods, a Cox proportional hazard (PH) model was also constructed wherein death was considered a censoring event and not a competing risk and included the same specification as the competing risks model. Bias of the Cox PH model HRs was calculated as the relative difference between the Cox HR and the competing risk HR and is reported as the percent bias. The one-year cumulative incidence of thrombotic events was reported for the total cohort by the Fine and Gray method for competing risks. One minus the Kaplan–Meier estimate was used to plot the cumulative probability of thromboembolism, ignoring the competing risk of death. All analyses were conducted using SAS version 9.4 (SAS Institute, Cary, NC, USA). The University of Kentucky Institutional Review Board approved the use of the data for this study.

## 3. Results

### 3.1. Incidence of Thrombosis

There were 1050 thrombosis events observed in 13,700 individuals during the one-year follow-up. This included 756 DVTs (72% of events), 238 PEs (22.7%), 51 ATs (4.9%), and 5 PVTs (0.5%). Nearly one-half (*N* = 520, 49.5% of events) occurred within the first 90 days after MM diagnosis. Table 1 includes the demographics and clinical characteristics of the cohort.

**Figure 1:**
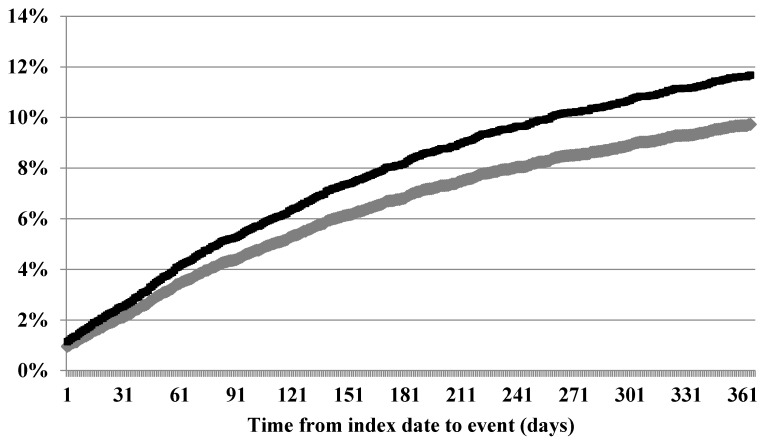
Comparison of Kaplan–Meier (black) and competing risk (gray) cumulative incidence functions of thromboembolic events.

The cohort contributed 9791.4 person-years of follow-up time for a one-year incidence rate of thrombosis of 107.2 (95% CI, 100.0–113.9) events per 1000 person-years. The highest incidence of thrombosis was in the first 30 days, with 251 events and an incidence rate of 234.2 (95% CI, 206.5–264.5) per 1000 person-years. The rate of thrombotic events decreased over the 60, 90, and 180 day intervals: 196.6 (95% CI, 178.3–216.4), 171.7 (95% CI, 157.4–187.0), 140.1 (95% CI, 130.5–150.1), per 1000 person-years, respectively, There were 384 deaths experienced as a competing risk and a total of 479 deaths during the study period with an incidence rate of 48.9 (95% CI, 44.7–53.5) deaths per 1000 person-years. The one-year cumulative incidence of thrombosis was 9.2% (95% CI, 8.7%–9.7%) for the total cohort (Figure 1) in the competing risk framework. Additionally, Figure 1 shows that the Kaplan–Meier method overestimates the cumulative incidence, which underlies the statistical models.

### 3.2. Survival Model Results

Table 2 demonstrates the bias introduced by using Cox PH models in the presence of competing risks. The highest bias can be observed in factors that would hypothetically be more associated with the omitted competing risk such as severe comorbidities and increasing age. For example, if competing risks are omitted, a much stronger association is observed between the 75+ age group and VTE, which represents a 75% bias over the competing risks estimate. Older age was associated with an increase in the hazard of thrombosis for the 35–64 and 65–74 age groups compared to the 18–34 reference group (Table 2). Female gender showed a protective effect with HR = 0.7 (95% CI, 0.7–0.8) compared to males. Increasing comorbidity burden had no impact on the hazard of thrombosis at baseline; however, some individual comorbidities at baseline did increase the risk. Those with CHF had 70% higher hazard (HR = 1.7 (95% CI, 1.4–2.1)) and those with hypertension had 20% higher hazard (HR = 1.2 (95% CI, 1.0–1.3)). Diagnosis with both leukocytosis (HR = 1.3 (95% CI, 1.0–1.9)) and leukopenia (HR = 1.6 (95% CI, 1.1–2.2)) had increased hazard of thrombosis in this population. There were no other significant associations observed for the other included covariates.

## 4. Discussion

Treatment advances over the last decade for MM have led to an increase in median survival greater than 5 years [34,35]. However, thrombotic complications have emerged as serious adverse effects of treatment driving the consideration of thromboprophylaxis in guidelines and RCTs in this patient population [36,37]. Despite the known risk, the pathogenesis of thrombosis in MM is poorly understood due to the various factors that can impart risk including patient characteristics, disease-related factors, as well as treatment-related risks [7]. Although thrombotic events have not been shown to have a large impact on overall survival specifically in MM [36], thrombosis events can cause interruption in therapy as well as tremendous economic and humanistic burdens in the MM population [38,39]. Recent American Society of Clinical Oncology guidelines have called for better evidence regarding the increased risk of thrombosis and MM so that prevention efforts can be focused towards periods of highest risk [40].

This study investigated two statistical approaches to assess the baseline factors related to thrombosis risk in newly diagnosed MM. For this population the competing risk of death is a contribution by many factors, including the advanced age of the cohort, having cancer, as well as the risk of death from the other outcome events [30]. The primary model used a competing risks framework, given that the outcome events cannot be considered independent of each other, *i.e.* experiencing one may preclude experiencing another or one event may cause another. Failure to do so can overestimate survival for traditional Kaplan–Meier or proportional hazard based analyses and lead to inflated cumulative incidence functions and biased associations [30]. In this study, this would have overestimated the cumulative incidence to be nearly 12% over the one-year study period. For measures of association in the regression models, the largest bias was observed for those factors that would be more highly associated with the competing risk. For example, age 75 and older had a HR of 2.8 (95% CI 1.6–4.8) in the Cox PH model. This is compared to the “true” value from the competing risk model of 1.6 (0.9–2.7)—a relative bias of 75%. Likewise, there was significant bias associated with liver disease (54.5%), dementia (25.0%), peptic ulcer disease (25.0%), and chronic pulmonary disease (25.0%). More importantly, as in the case of increasing age and peripheral vascular disease, the bias alters the bounds of the confidence interval to include the null making the interpretation of the findings more tedious.

In the primary analysis, there were few baseline factors found to be associated with risk in this study suggesting that risk may be associated with factors related to treatment of MM instead of pre-existing, patient-related factors. We observed that nearly one-half of all thrombotic events occurred within the first 90 days after MM diagnosis. A similar finding has been found by other studies, suggesting that the risk in the initial stage of diagnosis and treatment may be related to high tumor burden and release of thrombogenic factors with initiation of treatment [16,41,42]. This underscores the need to identify early risk factors at diagnosis to guide the utilization of thromboprophylaxis—especially in the first 90 days of treatment. In a *post*-*hoc* analysis restricting events to the first 90 days and in the full model analysis presented here, however, we found few demographic or clinical characteristics that were predictive of outcome events. This suggests that thrombosis risk in this population may be primarily driven by treatment and not necessarily any underlying risk. 

Due to the methodological focus and goals of this study, we ignored other factors related to VTE risk in multiple myeloma—mainly chemotherapeutic agents used and utilization of anticoagulant/antiplatelet therapy. It is well known that IMID therapy is associated with a large increase in risk and has led to thromboprophylaxis being commonplace for these patients. Treatment is, by nature, highly time-variant, given ever-changing treatment regimens as well as periods of treatment interspersed with periods of no treatment. This implies the usefulness of time-varying statistical frameworks and represents one of the primary limitations of competing risks analyses, given that competing risks methods are not compatible with time-varying covariates. While it is beyond the scope of this study to address time-varying exposures, we have shown the bias introduced when ignoring competing risks. Thus, there is a tenuous balance between including biased estimates of the survival functions or excluding time-varying covariates. Other methodologies can be utilized as well, such as the case-crossover or case-time-control study designs which avoid these setbacks. This study is subject to several limitations inherent to claims-based studies [43,44]. This study relied on ICD-9 coding available in the claims to diagnose study subjects with outcome events and comorbidities. It is impossible to confirm a positive diagnosis using these data; however, claims-based coding algorithms for VTE have been shown to perform strongly especially when there is a high risk of VTE in the population [32,45]. Further, information regarding MM severity and staging is not available in claims data and, thus, could not be included here. Likewise, medications not obtained using insurance, such as over-the-counter aspirin for thromboprophylaxis, cannot be observed in claims data and was unobserved in this study [46].

## 5. Conclusions

This study used a competing risks framework given that the outcome events cannot be considered independent of each other, *i.e.*, experiencing one may preclude experiencing another or one event may cause another. Failure to do so can overestimate survival for traditional Kaplan–Meier or proportional hazard based analyses and lead to inflated cumulative incidence functions and biased associations. In this study, this would have overestimated the cumulative incidence to be nearly 12% over the one-year study period. For this population the competing risk of death is a contribution by many factors including the advanced age of the cohort, having cancer, as well as the risk of death from the other outcome events. Death must be accounted for in older populations as well as in high mortality disease states when the incidence of death is high to avoid potential statistical pitfalls of traditional Kaplan–Meier and Cox PH methods.

## Figures and Tables

**Table 1:** Baseline characteristics of the cohort at diagnosis of multiple myeloma.

	Overall Cohort	
	*N* = 13,700	
**Age**	Mean 63.9	SD 13.7
18–34	283	2.1
35–64	7389	53.9
65–74	2648	19.3
75+	3380	24.7
**Gender**		
Male	6625	48.4
Female	7075	51.6
**Charlson Comorbidity Index**	Mean 1.1	SD 1.5
0	6892	50.3
1–2	4758	34.7
3–4	1476	10.8
5+	574	4.2
**Comorbidity**		
MI	251	1.8
CHF	933	6.8
PVD	841	6.1
Dementia	123	0.9
COPD	1817	13.3
Rheumatism	734	5.4
PUD	134	1.0
Mild liver disease	551	4.0
Diabetes	2814	20.5
Diabetes with complications	755	5.5
Paralysis	68	0.5
Renal disease	1826	13.3
Severe liver disease	45	0.3
CVD	910	6.6
HIV/AIDS	30	0.2
Hypertension	6466	47.2
CHD	1725	12.6
Lipids	4260	31.1
High platelets	70	0.5
High white cell	270	2.0
Anemia	3727	27.2
Obesity	491	3.6
Hypocoagulopathies	544	4.0
Thrombocytopenia	363	2.7
Low white cell	237	1.7
**Year of Diagnosis**		
2009	2469	18.0
2010	2476	18.1
2011	3153	23.0
2012	3053	22.3
2013	2549	18.6

CHF = congestive heart failure; PVD = peripheral vascular diseases; COPD = chronic obstructive pulmonary disease; PUD = peptic ulcer disease; CVD = cerebrovascular disease; CHD = coronary heart disease; SD = standard deviation.

**Table 2:** Results of competing risks model compared to the Cox PH model.

	Competing Risks			Cox PH			
Covariate	sHR	95% CI		HR	95% CI		% Bias
Age							
18–34	Ref.	Ref.	Ref.	Ref.	Ref.	Ref.	
* 35–64	1.7	1.0	3.0	2.2	1.3	3.7	29.4%
* 65–74	1.9	1.1	3.2	2.4	1.4	4.1	26.3%
75+	1.6	0.9	2.7	2.8	1.6	4.8	75.0%
Gender							
Male	Ref.	Ref.	Ref.	Ref.	Ref.	Ref.	
* Female	0.7	0.7	0.8	0.7	0.7	0.8	0.0%
Charlson Comorbidity Index							
0	Ref.	Ref.	Ref.	Ref.	Ref.	Ref.	
1–2	1.0	0.5	2.1	1.0	0.8	1.2	0.0%
3–4	0.8	0.5	1.3	0.8	0.6	1.2	0.0%
5+	1.0	0.8	1.2	0.9	0.5	1.7	−10.0%
Comorbidities							
MI	0.9	0.6	1.4	1.1	0.8	1.5	22.2%
* CHF	1.7	1.4	2.1	1.9	1.6	2.3	11.8%
PVD	1.2	0.9	1.5	1.2	1.0	1.4	0.0%
Dementia	1.2	0.7	2.1	1.5	0.9	2.3	25.0%
COPD	0.9	0.8	1.1	1.1	0.9	1.3	22.2%
Rheumatism	0.9	0.6	1.2	0.7	0.5	1.0	−22.2%
PUD	0.8	0.5	1.5	1.0	0.7	1.6	25.0%
Mild liver disease	0.8	0.5	1.1	0.9	0.7	1.2	12.5%
Diabetes	1.0	0.8	1.2	1.1	0.9	1.2	10.0%
Diabetes with complications	1.1	0.8	1.5	1.0	0.7	1.3	−9.1%
Paralysis	1.4	0.8	2.5	1.2	0.7	2.0	−14.3%
Renal disease	1.0	0.8	1.3	1.0	0.8	1.3	0.0%
Severe liver disease	1.1	0.4	3.0	1.7	0.9	3.4	54.5%
CVD	1.0	0.8	1.3	1.1	0.9	1.4	10.0%
* Hypertension	1.2	1.0	1.3	1.1	1.0	1.3	−8.3%
CHD	1.0	0.8	1.1	1.0	0.9	1.1	0.0%
Lipids	1.0	0.9	1.1	1.0	0.9	1.1	0.0%
High platelets	1.0	0.5	2.1	1.0	0.5	1.8	0.0%
* High white cell	1.3	1.0	1.9	1.2	0.9	1.6	−7.7%
Anemia	0.9	0.8	1.1	1.0	0.9	1.6	11.1%
Obesity	1.2	0.9	1.6	1.1	0.9	1.4	−8.3%
Hypocoagulopathies	0.9	0.6	1.4	0.8	0.6	1.3	−11.1%
Thrombocytopenia	1.0	0.6	1.8	1.2	0.8	2.0	20.0%
* Low white cell	1.6	1.1	2.2	1.4	1.0	1.9	−12.5%

sHR = subdistribution hazard ratio; CI = confidence interval; MI = myocardial infarction; CHF = congestive heart failure; PUD = peptic ulcer disease; CVD = cerebrovascular disease; CHD = coronary heart disease; Ref. = Reference category;* *p* < 0.05.

## Abbreviations

The following abbreviations are used in this manuscript:

VTE: venous thromboembolism
MM: multiple myeloma
IMID: immunomodulating drugs
PI: proteasome inhibitors
CVC: central venous catheters
LMWH: low-molecular weight heparin
RCT: randomized controlled trial
ICD-9-CM: International classification of diseases, 9th revision, clinical modification
DVT: deep vein thrombosis
PE: pulmonary embolism
HR: hazard ratio
PH: proportional hazard
CI: confidence interval
PVT: portal vein thrombosis
AT: arterial thrombosis
SD: standard deviation
